# Effects of exercise training on cardiovascular adrenergic system

**DOI:** 10.3389/fphys.2013.00348

**Published:** 2013-11-28

**Authors:** Dario Leosco, Valentina Parisi, Grazia D. Femminella, Roberto Formisano, Laura Petraglia, Elena Allocca, Domenico Bonaduce

**Affiliations:** Department of Translational Medical Sciences, University of Naples “Federico II,”Naples, Italy

**Keywords:** exercise, heart failure, adrenergic system, aging process, systemic hypertension

## Abstract

In heart failure (HF), exercise has been shown to modulate cardiac sympathetic hyperactivation which is one of the earliest features of neurohormonal derangement in this syndrome and correlates with adverse outcome. An important molecular alteration related to chronic sympathetic overstimulation in HF is represented by cardiac β-adrenergic receptor (β-AR) dysfunction. It has been demonstrated that exercise reverses β-AR dysfunction by restoring cardiac receptor membrane density and G-protein-dependent adenylyl cyclase activation. In particular, several evidence indicate that exercise reduces levels of cardiac G-protein coupled receptor kinase-2 (GRK2) which is known to be involved in both β1-AR and β2-AR dysregulation in HF. Similar alterations of β-AR system have been described also in the senescent heart. It has also been demonstrated that exercise training restores adrenal GRK2/α-2AR/catecholamine (CA) production axis. At vascular level, exercise shows a therapeutic effect on age-related impairment of vascular reactivity to adrenergic stimulation and restores β-AR-dependent vasodilatation by increasing vascular β-AR responsiveness and reducing endothelial GRK2 activity. Sympathetic nervous system overdrive is thought to account for >50% of all cases of hypertension and a lack of balance between parasympathetic and sympathetic modulation has been observed in hypertensive subjects. Non-pharmacological, lifestyle interventions have been associated with reductions in SNS overactivity and blood pressure in hypertension. Several evidence have highlighted the blood pressure lowering effects of aerobic endurance exercise in patients with hypertension and the significant reduction in sympathetic neural activity has been reported as one of the main mechanisms explaining the favorable effects of exercise on blood pressure control.

## Introduction

It has been generally accepted that regular physical activity is associated with beneficial effects on the cardiovascular system (Belardinelli et al., 1999; Hambrecht et al., 2000; Piepoli et al., 2004; Rinaldi et al., 2006; Flynn et al., 2009; Giallauria et al., 2013). In fact, the idea that exercise maintains cardiovascular health is evident by the direct links between a sedentary lifestyle and the risk of cardiovascular and other disease states. Cardiovascular diseases, such as heart failure (HF) and hypertension, and impairment of cardiovascular reserve observed with aging, are often associated with SNS overactivity (Francis and Cohn, 1986; Brodde et al., 1995a; Kaye et al., 1995; Davies et al., 1996; Julius and Nesbitt, 1996; Ferrara et al., 1997a,b; Xiao et al., 1998; Seals and Esler, 2000; Kilts et al., 2002; Schlaich et al., 2004). Conversely, exercise has been shown to decrease elevated SNS activity in HF, hypertension and in the aging heart and vasculature. Although somewhat controversial in humans, evidence from animal studies also indicates that exercise training reduces baroreflex-mediated and other forms of sympathoexcitation in normal individuals. Collectively, these data are consistent with the hypothesis that physical activity may decrease the incidence of cardiovascular disease and improve cardiac outcome via alterations in SNS activity. Despite the important clinical implications of this possibility, the mechanisms by which exercise alters control of SNS activity remain to be fully elucidated. The aim of this review is to focus on the pathophysiological mechanisms by which exercise can modulate SNS overactivity and exert its favorable effect on the onset and progression of cardiovascular diseases.

## Effects of exercise on SNS hyperactivity in heart failure

Sympathetic activation has been shown to be one of the earliest features of neurohormonal rearrangement in HF where the prolonged exposure to pathological levels of catecholamines (CAs) are associated with adverse outcome (Kaye et al., 1995; Marciano et al., 2012; Paolillo et al., 2013; Rengo et al., 2013a; Savarese et al., 2013). In this process, cardiac β-adrenergic receptor (β-AR) dysfunction seems to be crucial (Bristow et al., 1986; Ungerer et al., 1993; Brodde et al., 1995b; Xiao et al., 1999; Rockman et al., 2002; Giallauria et al., 2010; Rengo et al., 2012a; Femminella et al., 2013), in particular downregulation/desensitization of β1-AR, and prevalent desensitization/uncoupling of β2-AR. In particular, the receptors dysfunction seem to be related to increased levels of cardiac G-protein coupled receptor kinase-2 (GRK2). GRK2 is a kinase that phosphorylates intracellular domains of activated receptors, leading to the recruitment of arrestins to the receptors and the attenuation of intracellular G protein-dependent signaling. Therefore, GRK2 phosphorylates receptors and uncouples them from the adenylyl cyclase effector system (Rengo et al., 2012a). The relevance of cardiac GRK2 up-regulation in failing myocardium is supported by the observation of the therapeutic effect exerted by its inhibition (Salazar et al., 2013). Interestingly, GRK2 inhibition reverses left ventricular (LV) remodeling and improves myocardial contractility in the failing heart (Raake et al., 2008; Rengo et al., 2009, 2011, 2012a,b,c; Ciccarelli et al., 2011; Lymperopoulos et al., 2012).

Since it has been demonstrated that myocardial GRK2 levels and activity mirror those measured in peripheral lymphocytes in HF patients (Iaccarino et al., 2005), it could be possible to monitor the efficacy of different therapies using circulating white blood cells (Rengo et al., 2013b).

Exercise training in patients with stable HF, can relieve symptoms, improve exercise capacity and quality of life, and reduce disability, hospitalization, and mortality (Piepoli et al., 2004; van Tol et al., 2006). Physical inactivity can thus be considered a major cardiovascular risk factor and current treatment guidelines recommend exercise training in patients with HF in NYHA functional classes II and III (Hunt et al., 2005; Rinaldi et al., 2006). Exercise training is associated with numerous pulmonary, cardiovascular, and skeletal muscle metabolic adaptations that are beneficial.

The crucial role of β-AR dysregulation in the pathophysiology of HF is well established. GRK2, which plays a key role in the regulation of β-AR, is significantly elevated in human and experimental HF (Ungerer et al., 1993; Gros et al., 2000; Rockman et al., 2002; Petrofski and Koch, 2003; Iaccarino et al., 2005). Moreover, molecular manipulations of β-AR utilizing GRK2 inhibitors, such as the peptide known as the βARKct, restore β-AR signaling in the heart and increase cardiac function (Koch et al., 1995; Rockman et al., 1998; Harding et al., 2001; Shah et al., 2001). A significant reduction of cardiac GRK2 expression has been also recognized as a potential mechanism by which selective and non-selective β-AR blockade may positively affect β-AR signaling (Iaccarino et al., 1998; Leosco et al., 2007; Cannavo et al., 2013a; Rengo et al., 2013c). Previous works have shown that exercise is able to decrease GRK2 myocardial levels and improve β-AR signaling and responsiveness in spontaneously hypertensive rats (SHR) (MacDonnell et al., 2005) as well as in the aged heart (Leosco et al., 2007). More recently, it has been demonstrated that training evokes similar effects on β-AR system also in the post-ischemic hypertrophied failing myocardium leading to an enhanced cardiac inotropic state at the adrenergic stimulation (Leosco et al., 2008). Similar observation also have been reported in post-MI exercised mice (de Waard et al., 2007) that show increased cardiac β-AR protein and cAMP levels in the exercise animal group but these findings are not associated with significant changes of GRK2 protein levels. In this vein, it has been demonstrated that the mechanisms of β-AR desensitization may be GRK2- dependent (MacDonnell et al., 2005) or GRK2-independent (Xiao et al., 1994). Importantly, the observation of improved β-AR responses also in intact cardiomyocytes of post-infarcted failing hearts indicate that training may restore receptor signaling alterations in remote non-infarcted myocardium contributing to LV dysfunction (Leosco et al., 2008; Cannavo et al., 2013b). It still remains an unresolved issue why exercise does not seem to affect basal LV contractility in failing hearts (Musch et al., 1986; Gaudron et al., 1994) despite the improved β-AR function. Consistent with this is the observation that basal cAMP production, which remains still depressed in exercised HF animals, is unchanged, since adenylyl cyclase activity and cardiac contraction via protein kinase A mediated downstream effects are closely interlinked (Georget et al., 2002). This finding strongly supports the importance of downstream cellular events in the improvement of β-AR signaling and responsiveness in the failing heart.

## Effects of exercise on adrenal α2-ARs dysregulation in heart failure

As mentioned above, exercise training appears to reduce autonomic derangement and neurohumoral excitation at rest in HF. The effects of exercise training on adrenergic hyperactivation in HF patients have not been completely clarified. Recently, an important molecular mechanism has been identified that contributes to the sympathetic overdrive of the failing heart. This mechanism involves the upregulation of GRK2 in adrenal medulla of HF animals, which leads to downregulation and G protein uncoupling of the α2-ARs present in the chromaffin cell membranes of the adrenal gland that normally exert negative feedback control on CA turnover (Lymperopoulos et al., 2007a; Rengo et al., 2012a,b,c,d,e). Thus, dysfunction of these receptors results in chronically elevated CA secretion and circulating levels in HF (Lymperopoulos et al., 2007a). More recently, a novel molecular neurohormonal mechanism has been reported to explain the effects of exercise training on counterbalancing sympathetic overactivation and the enhanced circulating CA levels of chronic HF that significantly increase the morbidity and mortality of this devastating disease. This mechanism involves lowering and restoration of adrenal GRK2 levels/activity, which results in marked reduction of adrenal CA production and secretion via decreased adrenal α2-AR desensitization/downregulation and normalization of circulating CA levels (Rengo et al., 2010). This finding is of particular importance because several studies have reported that exercise training counteracts the catecholaminergic activation of chronic HF in humans and in several animal models of the disease (Gademan et al., 2007; Lymperopoulos et al., 2007a,b; Rengo et al., 2009); however, essentially no evidence has been provided to mechanisms mediating this beneficial effect of this modality in HF. Circulating CAs originate from the adrenal medulla in the form of Epinephrine and Norepinephrine, which are secreted at a ratio of 80–20%, respectively, under normal conditions (Lymperopoulos et al., 2007b). Spilled-over Norepinephrine produced at sympathetic nerve endings also contributes to the total circulating amount of CA. Adrenal CA production is under tight regulation by sympathoinhibitory α2-ARs, which are expressed in the adrenal medulla and inhibit CA release (Lymperopoulos et al., 2007b). α2-AR function in turn is regulated by GRK2, which phosphorylates and desensitizes the α2-AR, thus suppressing its function (Petrofski and Koch, 2003). By reducing GRK2 activity on adrenal α2-ARs, exercise training appears to restore adrenal α2-AR number and CA feedback inhibition, and this represents a mechanism whereby it reduces circulating CA levels in chronic HF.

## Effects of exercise on age-related cardiac and vascular β-AR dysregulation

Noteworthy, alterations of β-AR system, similar to those observed in HF, have been described also in the senescent heart (Davies et al., 1996; Ferrara et al., 1997a,b; Rengo et al., 2012a,b,c,d,e). With aging, sympathetic activity is increased and cardiac neuronal uptake of CA is decreased. Although alterations in Gs-coupled receptors in the failing and aging human heart are quite comparable, GRK2 activity seems to be not affected by age (Xiao et al., 1998). In this vein, animal studies demonstrated that the positive inotropic effects after both β1- and β2-AR stimulation are markedly decreased in myocardium of aged rats as a consequence of a lower density of receptors and a diminished adenylyl cyclase activity. There have been conflicting reports about the effect of age on cardiac inhibitory G protein (Gi) levels in both humans and rodents. In one study of human heart, Gi levels were measured in atrial appendages received from surgical patients, and it was found that Gi expression increased with age (Brodde et al., 1995a,b). Accordingly, age-dependent Gi upregulation has been documented in animal models. This observation is particularly relevant since β2-AR signaling couples to Gi proteins as well as to stimulatory G proteins (Gs) (Kilts et al., 2002). In contrast, some authors reported that neither GRKs nor Gi proteins appear to contribute to the age-related reduction in cardiac β-AR responsiveness (Xiao et al., 1998). This evidence can be the consequence of a delayed progression of sympathetic activity dysfunction in the elderly, while in HF it develops much more rapidly (Brodde et al., 1995a,b; Kilts et al., 2002). Thus, time course and intensity of increase in sympathetic activity can explain the different behavior of GRK activity in the aging and failing human heart.

Exercise has been shown to modulate GRK2 levels/activity by reducing levels of this kinase in the heart and, consequently, inducing β-AR “resensitization.” It has been previously demonstrated in rats that both exercise and β-blockers reverse β-AR dysfunction by restoring cardiac receptor membrane density and G-protein-dependent adenylyl cyclase activation (Leosco et al., 2007). Of note, although cardiac GRK2 levels were not upregulated in old sedentary rats compared to young sedentary rats, exercise resulted in a significant reduction of GRK2 activity even at lower levels than those observed in young controls. This latter phenomena represents a further demonstration of the beneficial effects of physical activity on β-AR signaling. Furthermore, Böhm et al. have demonstrated that exercise can partially reverse depression in cAMP production due to age-dependent Gi alpha increased expression (Böhm et al., 1993).

At vascular level, studies conducted in the aorta and carotid arteries of old rats have shown a reduced β-AR-dependent vasorelaxation (Chapman et al., 1999; Schutzer et al., 2001; Leosco et al., 2003). Importantly, β-AR dysfunction observed in the aorta and carotids of old rats is mainly due to GRK2 upregulation that seems to have a crucial pathogenic role in age-related vascular β-AR dysfunction. Importantly, exercise shows a therapeutic effect on age-related impairment of vascular reactivity to adrenergic stimulation and restores β-AR-dependent vasodilatation by increasing vascular β-AR responsiveness and by reducing endothelial GRK2 activity (Leosco et al., 2003).

In old healthy subjects, it has been demonstrated that physical training ameliorates age-related deterioration of cardiac function in terms of enhanced LV inotropic response to CA (Ehsani et al., 1991; Stratton et al., 1994; Spina et al., 1998). Contrasting data have been reported by other authors who described unchanged LV systolic performance (Stratton et al., 1992) in response to adrenergic stimulation after training in the elderly. However, it is important to underline that exercise training also enhances vagal tone (Levy et al., 1966), which could mask the favorable effect of exercise on cardiac β-adrenergic responsiveness.

## Effects of exercise on neural regulation of blood pressure

The most common form of hypertension is neurogenic hypertension that is associated with sympathetic overdrive, loss of parasympathetically mediated cardiac variability, and excessive angiotensin II activity (Esler, 2010). Evidence from studies in both patients and animal models of hypertension strongly implicate the chronic sympathetic neural activation in the etiology and progression of hypertension (Anderson et al., 1989; Smith et al., 2002, 2004; Simms et al., 2009; Esler, 2010). Studies in adult SHR have also identified a reduced cardiac parasympathetic nerve activity (Friberg et al., 1988), elevated SNS activity and increased noradrenaline release (Judy and Farrell, 1979; Lundin et al., 1984). Notably, neonatal sympathectomy prevents the SHR from developing hypertension (Cabassi et al., 1998), and SNS is elevated in young SHR prior to the development of hypertension (Korner et al., 1993). In humans, it has been estimated that a neurogenic component is observed in 40–65% of hypertensive patients and different studies report an increase of 100–200% of SNS activity in the brain, heart, kidneys, and skeletal muscle vasculature (Esler et al., 1988; Grassi et al., 1998a; Huggett et al., 2004; Lambert et al., 2007). The magnitude of the elevation in SNS is related to the magnitude of hypertension (Grassi, 1998; Grassi et al., 1998b). Indeed, it has been described that the increase in blood pressure from control subjects to mildly hypertensive, and to more severely hypertensive patients is associated with a parallel increase in muscle SNS activity (Grassi et al., 1998b).

Clinical interventions showing impressive blood pressure lowering effects by targeting reductions in SNS activation (Krum et al., 2009, 2011; Wustmann et al., 2009; Esler et al., 2010) have contributed to a better understanding of the central sympathetic regulatory pathways altered in hypertension, and have stressed the importance in the control of the sympathetic nervous system in hypertension and its utility as a clinical target. Exercise has been shown to reduce SNS hyperactivity and blood pressure in hypertension. There are important mechanistic data from animal studies to show that exercise training limits sympathoexcitation and favors sympathoinhibition in the brainstem cardiovascular centers (Mueller, 2007). Previous studies have highlighted the blood pressure lowering effects of aerobic endurance exercise training in patients with hypertension (Esler et al., 2010). Indeed, regular, moderate intensity training is associated with a 10 mmHg average fall in systolic and diastolic blood pressure in hypertensive patients (American College of Sports Medicine, 1993). In this regard, it has been reported that a 4-month programme of aerobic exercise training reduces of ~37% muscle SNS activity and blood pressure in never-treated hypertensive patients (Laterza et al., 2007).

Exercise training could elicit adaptations in the adrenergic system, since SNS is activated during each bout of exercise and repeated activation of SNS may result in an attenuation of sympathetic activity (Grassi et al., 2000). Animal studies suggested that nitric oxide decreased overall sympathetic excitability within the brainstem and possibly through actions in higher brain regions (Goodson et al., 1994; Patel et al., 1996). It is unclear whether the increased release of nitric oxide during exercise training has a central sympathoinhibitory effect in humans. Previous studies demonstrated that hyperinsulinemia and insulin resistance were associated with hypertension and sympathetic activation (Julius et al., 1991; Baron et al., 1993). Training-dependent improvement of insulin sensitivity in normotensive and hypertensive individuals (Kohno et al., 2000; Henriksen, 2002) could contribute to attenuate insulin mediated sympathetic activation.

## Conclusions

SNS overactivity is common in many cardiovascular disease states and is related to a higher incidence of morbidity and mortality. It is widely accepted that exercise training is associated with reductions in SNS activity, whether at rest or during conditions that produced sympathoexcitation, and this effect may represent an important mechanism by which exercise may contribute to long term cardiovascular health. Future studies are needed to further identify the molecular mechanisms that are involved in physical activity dependent changes in the control of SNS activity.

### Conflict of interest statement

The authors declare that the research was conducted in the absence of any commercial or financial relationships that could be construed as a potential conflict of interest.
